# Analogous Anti-Ferroelectricity in Y_2_O_3_-Coated (Pb_0.92_Sr_0.05_La_0.02_)(Zr_0.7_Sn_0.25_Ti_0.05_)O_3_ Ceramics and Their Energy-Storage Performance

**DOI:** 10.3390/ma12010119

**Published:** 2019-01-01

**Authors:** Bijun Fang, Yan Jiang, Xiangyong Zhao, Shuai Zhang, Dun Wu, Jianning Ding

**Affiliations:** 1 School of Materials Science and Engineering, Jiangsu Collaborative Innovation Center of Photovolatic Science and Engineering, Jiangsu Province Cultivation Base for State Key Laboratory of Photovoltaic Science and Technology, National Experimental Demonstration Center for Materials Science and Engineering, Changzhou University, Changzhou 213164, China; yan_jiang@cczu.edu.cn (Y.J.); shuaizhang@cczu.edu.cn (S.Z.); wudun@cczu.edu.cn (D.W.); 2 Key Laboratory of Optoelectronic Material and Device, Department of Physics, Mathematics & Science College, Shanghai Normal University, Shanghai 200234, China; 3 School of Material Science and Engineering, Jiangsu University, Zhenjiang 212013, China

**Keywords:** PSLZSnT antiferroelectric-like ceramics, self-combustion method, perovskite structure, oxide doping, energy-storage density

## Abstract

Antiferroelectric analogous (Pb_0.92_Sr_0.05_La_0.02_)(Zr_0.7_Sn_0.25_Ti_0.05_)O_3_ (PSLZSnT) ceramics were prepared by the solid-state sintering method by introducing a Y_2_O_3_-coating via the self-combustion method. The synthesized Y_2_O_3_-doped PSLZSnT ceramics present pseudo-cubic structure and rather uniform microstructural morphology accompanied by relatively small grain size. Excellent energy-storage performance is obtained in the Y_2_O_3_-doped PSLZSnT ceramics, in which the value of the energy-storage density presents a linearly increasing trend within the electric field measurement range. Such excellent performance is considered as relating to the rather pure perovskite structure, high relative density accompanied by relatively small grain size, and the antiferroelectric-like polarization-electric field behavior.

## 1. Introduction

With the development of miniature and compact electronic and electrical devices, and concerning sustainable development of society, designing for high power and energy-storage density dielectric materials becomes an urgent requirement [1,2]. Existing dielectric ceramics provide potentiality for high-energy-storage applications due to the fast charge/discharge capability, mature ceramic technique, and diversified composition selection, in which high dielectric constant, high dielectric breakdown strength, large polarization, and low hysteresis loss are desirable [2,3,4].

Antiferroelectric materials have gained continuous attention due to their double ferroelectric hysteresis loop characteristics and low dielectric loss, which tend to exhibit superior energy-storage density as compared with their ferroelectric counterparts [5,6,7]. Among which, PbZrO_3_ (PZ) was the first identified antiferroelectric compound, whereas the double polarization-electric field (P-E) hysteresis loop could not be driven at room-temperature in the polycrystalline ceramic state PZ since the critical electric field value (E_F_) induced the antiferroelectric to ferroelectric phase transition, exceeding the dielectric breakdown strength (E_b_), therefore, PZ presented no promising applications in engineering devices [5,7].

It is well known that the E_b_ value depends on interfacial polarization and grain boundary density, which correlate with point charge defects and grain size, respectively [2,7,8,9]. Chemical doping provides an effective way to tailor physical performance, in which Sn and Y doping are reported as efficient in reducing the E_F_ value, relating to the incommensurate modulation of the lattice cell [10,11,12]. Y doping also greatly decreases grain size of the (Pb_0.87_Ba_0.1_La_0.02_)(Zr_0.65_Sn_0.3_Ti_0.05_)O_3_ (PBLZST) ceramics as compared with the undoped PBLZST ones, which can increase the E_b_ value and energy-storage density [12]. Appropriate addition of additional PbO can compensate the evaporation of PbO during sintering, inhibit the formation of the pyrochlore phase, and stabilize the double ferroelectric hysteresis loop of antiferroelectrics [13].

Based on the above considerations, the (Pb_0.92_Sr_0.05_La_0.02_)(Zr_0.7_Sn_0.25_Ti_0.05_)O_3_ (PSLZSnT) ceramics were prepared by the traditional ceramic processing by introducing Y_2_O_3_-coating via the self-combustion method [14]. Forming a solid-solution by adding SrTiO_3_ and PbTiO_3_ stabilizes the perovskite structure, modifies the E_F_ value, and then improves the antiferroelectric P-E hysteresis loop [2,5,7]. Introducing the Y_2_O_3_-coating via the self-combustion method was to thoroughly utilize its effect on inhibiting grain growth [12] since materials preparation processing presents essential influences on the crystal structure and materials’ performance [4,8,13,15,16]. The effects of the self-combustion technique and Y_2_O_3_ doping on the phase structure and electrical properties of the PSLZSnT ceramics were reported, and the electrical energy-storage performance was discussed.

## 2. Experimental Procedure

The Y_2_O_3_-doped (Pb_0.92_Sr_0.05_La_0.02_)(Zr_0.7_Sn_0.25_Ti_0.05_)O_3_ (PSLZSnT) ceramics were prepared by the solid-state sintering method by introducing Y_2_O_3_-coating via the self-combustion method. Stoichiometric oxides and carbonate of PbO, SrCO_3_, La_2_O_3_, ZrO_2_, SnO_2_, and TiO_2_ were weighed and well mixed according to the formula, in which an additional 5 mol% PbO was added to stabilize the antiferroelectric structure [13]. The mixture was calcined at 900 °C for 3 h and rather pure perovskite structure was obtained. Y_2_O_3_-coated PSLZSnT powder was synthesized via a self-combustion method, using Y(NO_3_)_3_⋅6H_2_O, citric acid, and C_19_H_42_BrN (CTAB, hexadecyl trimethyl ammonium bromide or cetyltrimethylammonium bromide), and the calcined PSLZSnT precursor powder as raw materials, and then self-combusted at 300 °C for 2 h as reported elsewhere [14]. The self-combusted Y_2_O_3_-coated PSLZSnT powder was cold-pressed into pellets after grinding and granulation, and sintered at 1275 °C for 2 h to prepare ceramics.

Crystal structure and microstructural morphology were characterized by XRD and SEM equipment (Rigaku D/max-2500/PC and Hitachi S-4800) (Akishima-shi, Tokyo, Japan and Chiyoda-ku, Tokyo, Japan), respectively. The fired-silver electrode was manually coated on both large surfaces of the sintered ceramics after polishing for physical performance measurements. The energy-storage properties were calculated using the P-E hysteresis loops measured by Radiant Precision Premier LC (Albuquerque, New Mexico, USA) [4,16].

## 3. Results and Discussion

**Figure 1:**
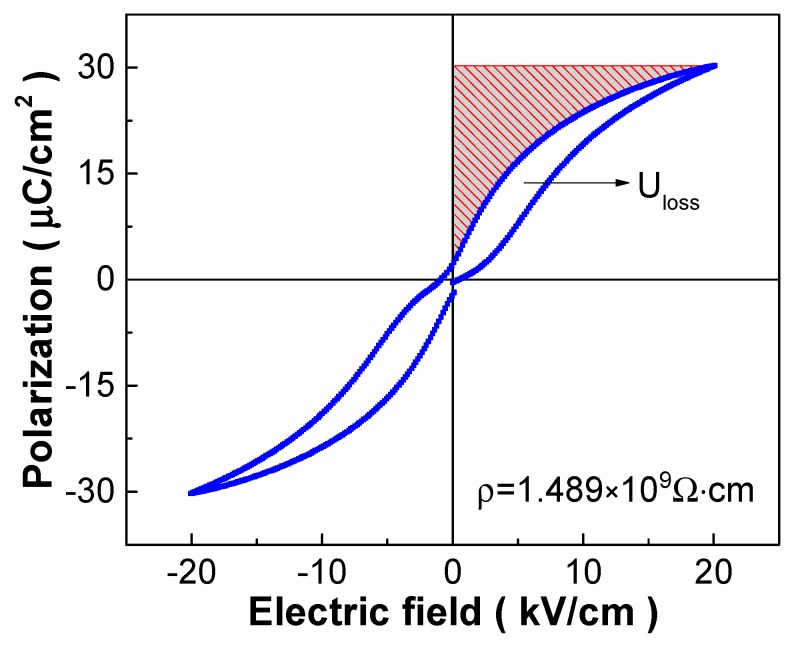
Polarization-electric field (P-E) hysteresis loop of the 0.25 mol% Y_2_O_3_-doped PSLZSnT ceramics. Inset shows resistivity and the shaded area with oblique lines shows the energy-storage density.

The energy-storage density is an important performance parameter for high-energy-storage dielectric capacitive applications [2,5,7]. Figure 1 shows the room-temperature P-E hysteresis loop of the 0.25 mol% Y_2_O_3_-doped PSLZSnT ceramics, based on which the energy-storage density U_C_ is calculated by the equation $U_{C}=\int EdD$ via integrating the effective shaded area accompanied by the energy-storage efficiency η calculated by the equation $\eta =\frac{U_{C}}{U_{C}+U_{loss}}$ [1,2,7]. The waist shrunk P-E loop can be observed clearly although no typical antiferroelectric double P-E loop is driven, i.e., the nonzero remnant polarization P_r_ value, which exhibits antiferroelectric analogous characteristics and presents a great difference between the P_r_ and saturation polarization P_s_ values. Such a big difference indicates that the electric displacement D will not saturate quickly with an increase of the external electric field, which is especially favorable to excite large energy-storage density and high energy-storage efficiency [1,2,7]. In this work, large energy-storage density and high energy-storage efficiency can be excited at only 20 kV/cm, being 176.6 mJ/cm^3^ and 66.09%, respectively, providing a possibility for prospective applications.

**Figure 2:**
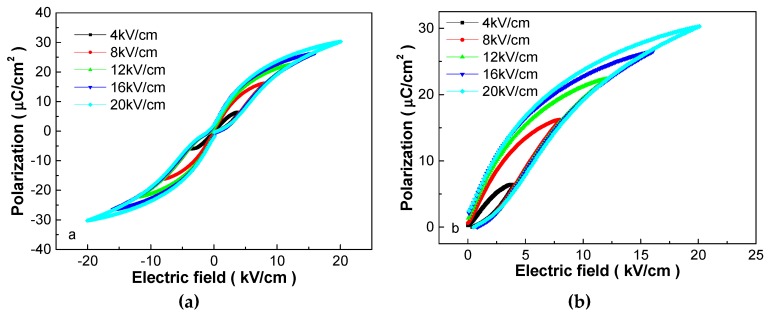
P-E hysteresis loops of the 0.25 mol% Y_2_O_3_-doped PSLZSnT ceramics measured at different electric fields. (**a**) bipolar; (**b**) unipolar.

For energy-storage applications, a high electric field is desirable, therefore, Figure 2 shows the P-E hysteresis loops of the 0.25 mol% Y_2_O_3_-doped PSLZSnT ceramics measured at different electric fields. With elevation of the external electric field, slim and antiferroelectric analogous hysteresis loops are maintained, whereas, the polarization and the difference between P_r_ and P_s_ increases greatly. Furthermore, the P-E hysteresis loop is far from saturation under 20 kV/cm, although a larger electric field was not undertaken due to the limitation of the equipment and the consideration of dielectric breakdown. Such phenomena are particularly beneficial for obtaining high energy-storage performance dielectrics [1,2,7]. Derived from Figure 2a, the P-E loops in one quadrant is presented in Figure 2b. Based on the magnified P-E loops, the parameters correlating with the energy-storage applications can be calculated.

**Figure 3:**
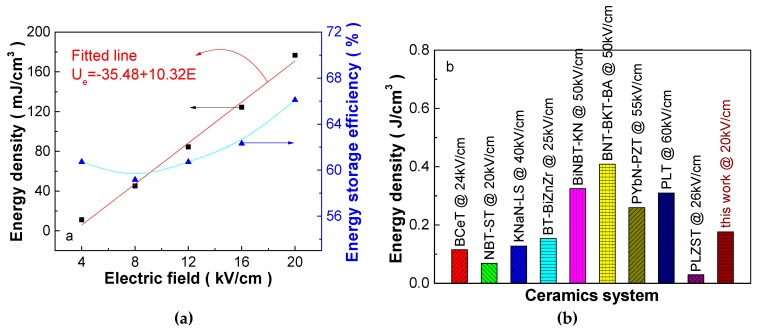
(**a**) Variation of energy-storage density and energy-storage efficiency as a function of electric field of the 0.25 mol% Y_2_O_3_-doped PSLZSnT ceramics; (**b**) comparison of energy-storage performance of some recently reported ferroelectric and antiferroelectric ceramics, in which BCeT is BaCe_0.15_Ti_0.85_O_3_ [20]; NBT-ST is 0.7(Na_0.5_Bi_0.5_)TiO_3_-0.3SrTiO_3_ [21]; KNaN-LS is 0.97(K_0.5_Na_0.5_)NbO_3_-0.03LiSbO_3_ [22]; BT-BiZnZr is 0.9BaTiO_3_-0.1Bi(Zn_0.5_Zr_0.5_)O_3_ [23]; BiNBT-KN is 0.94Bi_0.47_Na_0.47_Ba_0.06_TiO_3_-0.06KNbO_3_ [24]; BNT-BKT-BA is 0.94(0.75Bi_0.5_Na_0.5_TiO_3_-0.25Bi_0.5_K_0.5_TiO_3_)-0.06BiAlO_3_ [25]; PYbN-PZT is 0.075Pb(Yb_1/2_Nb_1/2_)O_3_-0.925Pb(Zr_0.36_Ti_0.64_)O_3_ [17]; PLT is (Pb_1-x_La_x_)Ti_1-x/4_O_3_ (x = 0.28) [26]; and PLZST is Pb_0.94_La_0.04_[(Zr_0.7_Sn_0.3_)_0.9_Ti_0.1_]O_3_ [19].

Figure 3a shows energy-storage density and energy-storage efficiency of the 0.25 mol% Y_2_O_3_-doped PSLZSnT ceramics under different electric fields. The energy-storage density presents a linearly increasing trend within the electric field measurement range, affording promising high-performance energy-storage applications. It is noteworthy that the energy-storage density tends to present an exponentially increasing trend under a high electric field, as reported in our previous work, in which the $\exp^{\left(E/5.9193\right)}$ relationship is excited above 40 kV/cm in the 0.075Pb(Yb_1/2_Nb_1/2_)O_3_-0.925Pb(Zr_0.36_Ti_0.64_)O_3_ ceramics [17] prepared by the conventional ceramic processing via the B-site oxide mixing route [18], indicating larger energy-storage density possibility. Such an increasing trend is not a rare phenomenon, such as the Pb_0.94_La_0.04_[(Zr_0.7_Sn_0.3_)_0.9_Ti_0.1_]O_3_ ceramics prepared by the solid-state reaction method reported by Xu et al., where apparent exponential elevation of the energy-storage density is observed above 60 kV/cm [19]. The energy-storage efficiency of the 0.25 mol% Y_2_O_3_-doped PSLZSnT ceramics decreases slightly at 8 kV/cm and increases greatly with a further increasing electric field, and the η value is larger than 60%, which can decrease energy dissipation during energy-storage cycling.

Figure 3b shows energy-storage performance of some lead-based and lead-free ferroelectrics and antiferroelectrics [17,19,20,21,22,23,24,25,26]. Considering the electric field used in this work, the energy-storage density of the 0.25 mol% Y_2_O_3_-doped PSLZSnT ceramics is far larger than these materials, and the energy-storage efficiency exceeds most of these materials. Furthermore, the waist shrunk P-E loops are often reported, such as in NBT-ST [21], BNT-BKT-BA [25], and PLZST [19], whether or not nominated as antiferroelectrics, the narrowed P-E loops are observed, and no apparent double ferroelectric loops are excited. The antiferroelectric-like behavior becomes evident with increasing temperature in NBT-ST, which can be attributed to the nominal ferroelectric to antiferroelectric phase transition [21]. The linear increase of energy-storage density with an increasing electric field under low electric field in NBT-ST [21] and BNT-BKT-BA [25], and the exponential increase of energy-storage density with increasing electric field under high electric field in PLZST [19], corroborate our anticipation that the Y_2_O_3_-doped PSLZSnT ceramics could present high-performance energy-storage properties.

**Figure 4:**
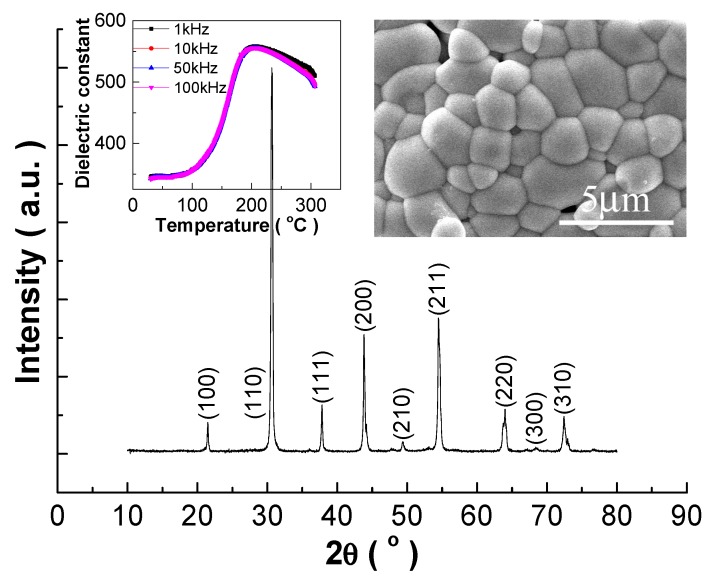
XRD pattern of the 0.25 mol% Y_2_O_3_-doped PSLZSnT ceramics. Inset shows temperature dependence of dielectric constant measured at several frequencies upon the cooling process and SEM image of the free surface after thermal etching at 850 °C for 30 min.

The high energy-storage performance obtained in this work can be attributed to crystal and morphology factors as shown in Figure 4. A rather pure perovskite structure is obtained, i.e., without detectable impurity within the detection limitation of the XRD measurement. Overall, the diffraction reflections are rather symmetric and present a single peak besides the {200} and {310} diffraction reflections, which present slight splitting. The distortion from the cubic symmetry is apparent, and the Y_2_O_3_-doped PSLZSnT ceramics may exist in a rhombohedral, tetragonal, or orthorhombic system structure. However, due to the sensitivity limitation of the conventional XRD measurement, accurate crystal refinement cannot be obtained, therefore, the pseudo-cubic perovskite structure is used for calculating the crystal cell parameters. The maximum dielectric constant value of 558 at 207.3 °C and 1 kHz and the rather broad dielectric peaks without apparent dielectric frequency dispersion coincide with the antiferroelectric-like dielectric behavior [2,5,7]. The grains distribute rather homogenously without apparent gas pores, considering the free-surface-sample used, and the average grain size is determined as 2.09 μm, made statistically by SmileView software, smaller than normal Pb-based ferroelectrics [4].

Although the 0.25 mol% Y_2_O_3_-doped PSLZSnT ceramics present excellent energy-storage performance under a low electric field, an exceptionally large external electric field is required for practical dielectric capacitor applications [2,5,7]. Polymer materials tend to present a high E_b_ value, allowing large energy-storage density to be obtained, such as in the biaxially-oriented polypropylene reported by Chu et al., in which ~4 J/cm^3^ energy-storage density was acquired under 6000 kV/cm [1]. It could be predicted that such a value can increase to ~53 J/cm^3^ of the Y_2_O_3_-doped PSLZSnT ceramics even in the linearly increasing trend with an increase of the external electric field under a large E_b_ value. Furthermore, non-early saturation of electric displacement and low dielectric loss are additional necessary parameters for high energy-storage applications disclosed by Chu et al. [1], which have been the essential properties of the Y_2_O_3_-doped PSLZSnT ceramics.

Then, the major task for the Y_2_O_3_-doped PSLZSnT ceramics is to increase the E_b_ value to meet the requirements of energy-storage applications, which correlates with the ceramics’ density, grain size, and defect chemistry [2,5,6,7]. Since polymer materials tend to show large E_b_ values, polymer-based dielectrics, especially in inhomogenous structures, provide an efficient strategy to increase energy-storage performance, i.e., forming polymer matrix composites which can combine the advantages of a high dielectric constant of ferroelectric ceramics and large E_b_ values of polymers [2,5,6,7]. Among which, polyvinylidene fluoride (PVDF)-based polymers attract great research attention due to their large dielectric constant in the ferroelectric state. Furthermore, chemical functionalization of ferroelectric ceramic precursor powders with improving distribution and overcoming interfacial polarization are key problems in forming polymer-based composites [2,5,7].

Although the Y_2_O_3_-doped PSLZSnT ceramics present a high relative density of ~95% ($\mathrm{relative}\;\mathrm{density}=\frac{\mathrm{bulk}\;\mathrm{density}}{\mathrm{theoretical}\;\mathrm{density}}\times 100\%$, in which the bulk density of the ceramics was measured by the Archimedes’ water immersion method, and the theoretical density of the ceramics was calculated based on the crystal cell volume obtained by the XRD result combined with the molecular weight) with a relatively small mean grain size of 2.09 μm, as compared with the normally synthesized Pb-based ceramics via the solid-phase method [4], further increase of the density with decreasing grain size is required to enhance energy-storage performance. Such requirements encounter dilemmas since relative density and grain size change contrarily if just tailoring sintering temperature, i.e., normally, relative density increases with elevating sintering temperature within an appropriate sintering temperature range, and inevitably leads to the growth of grain size [4].

Oxide coating introduced via the self-combustion method used in this work offers the possibility of tailoring these parameters simultaneously. Great progress is obtained in synthesizing 0.55Pb(Ni_1/3_Nb_2/3_)O_3_-0.05PbHfO_3_-0.4PbTiO_3_/Ni_0.875_Zn_0.125_Fe_2_O_4_ (PNNHT/NZF) particulate composite ceramics in our previous work [14]. Densified PNNHT/NZF ceramics are prepared sintered at only 900 °C by simultaneously introducing nano-sized WO_3_ and CuO coatings via the self-combustion method, which guarantees a small grain size due to the low sintering temperature [14]. An additional promising technique is adding glass-like sintering aids, which can increase the relative density of ceramics sintered at low sintering temperatures, beneficial for obtaining a small grain size. Glass-based inhomogenous dielectrics provide another alternative choice, in which the barium boroaluminosilicate glass (SchottAF-45) is proved to exhibit the highest E_b_ value of 12 MV/cm in a bulk glass state as reported by Smith et al. [27], whose dielectric breakdown strength is maintained as 1300 kV/cm and charge-discharge efficiency is increased to 92.5%, as reported by Xue, when mixed with 40 mol% (4BaO-Na_2_O-5Nb_2_O_5_) to form niobate glass-ceramics and diminished interfacial polarization [28].

Defect chemistry is also a problem that should be considered, influencing interfacial polarization [2,5,7]. In this work, Y_2_O_3_ doped into PSLZSnT will produce donor point defects although the charge balance is taken into account in chemical formulation design, then Pb vacancies are engendered to compensate charge balance, i.e., $Y_{2}O_{3}\overset{PSLZSnT}{\to }2Y_{Pb}^{\cdot }+V_{Pb}^{\prime \prime }+Pb↑+3O_{O}^{\times }$ considering cationic radii, expressed by the Kröger–Vink symbol system [29,30,31]. Such point defects are normally thermal-activated hopping-jumping charge carriers [31], tending to decrease resistivity of the sintered ceramics, especially at elevated temperatures. Resistivity of the 0.25 mol% Y_2_O_3_-doped PSLZSnT ceramics is 1.489 × 10^9^ Ω⋅cm in this work, as shown in Figure 1, which should be increased further to obtain a large E_b_ value required for high energy-storage density, in which heterovalent ion doping with self-charge-compensation combinations provides a promising strategy [32,33].

Based on the above discussions, formation of polymer- or glass-based composites can increase the E_b_ value; introducing nano-sized sintering aids can increase relative density meanwhile maintaining a small grain size due to low sintering temperatures; and self-charge-compensation heterovalent ion doping can diminish point defects, which provide effective methods to obtain high-performance energy-storage properties.

## 4. Conclusions

Y_2_O_3_-doped (Pb_0.92_Sr_0.05_La_0.02_)(Zr_0.7_Sn_0.25_Ti_0.05_)O_3_ (PSLZSnT) ceramics were prepared by the conventional ceramic processing by introducing Y_2_O_3_-coating via the self-combustion method. The Y_2_O_3_-doped PSLZSnT ceramics exhibit a rather pure pseudo-cubic perovskite structure. Their dielectric-temperature curves are slightly broad without obvious dielectric frequency dispersion, accompanied by several hundreds of relative dielectric constants and a small dielectric loss tangent. The sintered ceramics present rather homogenous microstructural morphology with a relatively small mean grain size of 2.09 μm. The Y_2_O_3_-doped PSLZSnT ceramics present antiferroelectric analogous behavior with waist shrunk P-E loops, where the P_r_ and P_s_ values exhibit a great difference. Such characteristics are favorable for energy-storage properties, therefore, large energy-storage density and high energy-storage efficiency were obtained, being 176.6 mJ/cm^3^ and 66.09%, respectively, and excited at only 20 kV/cm, which exceeds many recently reported lead-based and lead-free ferroelectric and antiferroelectric ceramics. To improve the energy-storage properties further, forming polymer- or glass-based composites, introducing nano-sized sintering aids, and self-charge-compensation heterovalent ion doping provide effective strategies.
